# Plasma Nanoengineering and Nanofabrication

**DOI:** 10.3390/nano6070122

**Published:** 2016-06-23

**Authors:** Krasimir Vasilev, Melanie Macgregor Ramiasa

**Affiliations:** 1 School of Engineering, University of South Australia, Adelaide, SA 5095, Australia; 2 Future Industries Institute, University of South Australia, Adelaide, SA 5095, Australia; melanie.ramiasa@unisa.edu.au

With the recent advances in nanotechnology, plasma nanofabrication has become an exciting new niche because plasma-based approaches can deliver unique structures at the nanoscale that cannot be achieved by other techniques and/or in a more economical and environmentally friendly manner. Over the last decade, there have been exiting breakthroughs in the utilization of plasma processes in the fabrication of a rich diversity of nanomaterials and nanoengineered coatings. Examples include nanowires, nanotubes, nanoparticles, and nanotextured coatings. Some of these materials can simply not be derived by conventional means while many others have remarkable plasma-induced properties setting them apart from their traditional analogue. These materials are revolutionizing a spectrum of fields, ranging from electronics to biology and medicine.

The goal of this Special Issue is to present some of the latest advances in the fields of plasma assisted nanoengineering and nanofabrication and their application to modern technologies. In addition, the Special Issue aims at highlighting current challenges and obstacles that lie on the path to fully understand the fundamental physical phenomena underlying the plasma facilitated fabrication of nanomaterials. Further, this Special Issue intends to provide guidance to researchers in the field and inform the community of exciting future directions.

This special issue contains a blend of 11 original research papers, communications and review type contributions from leading investigators in the field of plasma nanofabrication from across the world. The inspiring review by Puliyalil and Cvelbar [1] focuses on the use of plasma etching for the production of advanced polymeric materials for the fields of microelectronic, photonics, water repellence and medical devices. It provides a comprehensive synopsis of the mechanism governing the selective plasma etching of polymeric materials and will without a doubt inspire new research towards the next generation of plasma-derived nanomaterials. Several other quality research articles published in this special issue also focus on plasma etching and surface nanotexturing. For Instance, Fiflis et al. [2] produced nanotexture palladium surface using helium plasma. These advanced material substrates exhibit great integrity and better catalytic properties than their smooth counterparts. In the work of Mangla et al. [3], a dense plasma focus device was used to produce, for the first time, III–V semiconductors made of continuous and porous nano gallium arsenide (GaAs) composites. The interesting optical properties of these nanotextured materials (strong photoluminescence and high transmission) show potential for applications as transmission type photocathodes or in visible optoelectronic devices.

This special issue also contains articles presenting a novel plasma based approach for the synthesis of nanomaterials. Li et al. [4] report on the first fabrication and characterisation of titanium alloy composite nanoparticles prepared via hydrogen plasma-metal reaction. Ananth and Mok [5] used gentle, non-thermal dielectric barrier discharged (DBD) atmospheric plasma to produce silver and ruthenium oxide nanocomposite. The materials prepared with this environmentally friendly low energy plasma method display high crystallinity and good thermal resistivity. Tanaka et al. [6] investigated the formation of nanoparticles by induction thermal plasma for four different lithium composite systems. This systematic study reveals the influence of melting temperature and boiling point of the source materials on the nucleation, growth and shapes of the nanoparticles produced.

Another important aspect of plasma based nanomaterials, is to be able to homogeneously coat nanoparticles to control their outer surface properties. This topic is tackled in this special issue by Post et al. [7] who describe a way to achieve homogeneous coating on metal nanoparticles and agglomerates using post-DBD.

Two articles in this special issue are particularly focused on the practical applications of plasma derived nanomaterials and nanoengineered surfaces. Atmospheric pressure argon plasma was used to produce both silver and gold nanoparticles on tin oxide solar cells. In this application focused study, Dao and Dhoi [8] demonstrated that the plasma fabricated nanoparticles can tune the efficiency of the solar cells. Lai et al. [9] use argon plasma treated zinc oxide nanowire to improve the resistive switching of single nanowire-based memory cells. The findings of this study will help the development of new-age, small size and transparent, resistive random access memory storage media.

This special issue also presents two modelling studies of plasma facilitated processes for the fabrication of nanomaterials. First, Xiong et al. [10] investigated, from a theoretical point of view, the effect of key parameters such as droplet size, injection angle and velocity on nanoparticle processing in suspension plasma spray. The 3 dimensional model presented is validated with published experimental data and can be used to predict the nanoparticle suspension behaviour in novel spray coating technologies. Shigeta and Watanabe [11] took a computational approach to model the effect of saturation pressure on the growth process and size-composition distribution of metal-silicide nanoparticles produced via thermal plasma. This study provides valuable theoretical insights on the very complex processes involved in the growth mechanisms of nanopowders.

Finally, the Editorial team would like to thank all contributing authors for making this Special Issue a success.
